# Nudging in Animal Disease Control and Surveillance: A Qualitative Approach to Identify Strategies Used to Improve Compliance With Animal Health Policies

**DOI:** 10.3389/fvets.2020.00383

**Published:** 2020-08-05

**Authors:** Maria Garza, Estelle C. C. Ågren, Ann Lindberg

**Affiliations:** ^1^Veterinary Epidemiology, Economics and Public Health Group, Department of Pathobiology and Population Sciences, Royal Veterinary College, Hatfield, United Kingdom; ^2^Department of Disease Control and Epidemiology, National Veterinary Institute, Uppsala, Sweden

**Keywords:** animal health, disease control program, surveillance, decision making, behavioral economics, nudge theory, Europe

## Abstract

Professionals from seven European countries were interviewed to identify strategies used in the surveillance and control of animal infections to influence behaviors such as program enrollment, adoption of biosecurity measures, and engagement in surveillance. To find strategies that were well-designed from a theoretical perspective, three frameworks from nudge theory were applied to the strategies: the Nuffield ladder to determine the strength of the interventions, EAST to identify attributes of the strategies, and MINDSPACE to identify the psychological mechanisms involved. We found that almost two thirds (91/120) of the strategies were designed in a manner likely to trigger multiple psychological mechanisms, which is in line with the existing recommendations for successful effect, i.e., achieving a desired behavior. This was despite that the design of the strategies was based on professionals' empirical understanding of the requirements to achieve anticipated outcomes rather than the systematic use of methods from the behavioral sciences and psychology. The most commonly used strategy was provision of information, and the least used mechanism was making a desired behavior easy to perform. The findings in this study, with all the examples of strategies used, can serve as inspiration for others. The theoretical frameworks may also be beneficial to apply as a complement in future design of new strategies. This study did not include evaluation of how efficient different strategies have been, which would be an interesting area for future studies.

## Introduction

Nudge theory is a concept in behavioral science, political theory, and economics which argues that positive reinforcement and indirect suggestions to achieve non-forced compliance can influence the motives, incentives, and decision making of groups and individuals as effectively as direct instruction, legislation, or enforcement (1). It has previously been used in other fields, for example to promote environmentally sustainable behavior (2) and to help implement public policies (3), but its usefulness in supporting animal health policies is still to be explored (4).

Animal health surveillance and control activities are usually designed with the assumption that all actors are fully compliant, meaning there may be assumptions regarding sample sizes and risk behavior that are unrealistic. Deviations from these assumptions, i.e., non-compliance, may impact on the cost-effectiveness of risk-based surveillance strategies, where loss of information from high-risk population strata may significantly reduce surveillance sensitivity. It may also lead to underestimation of the introduction risks or detection probabilities.

Activities where participation is voluntary or requires complex actions are the most vulnerable to non-compliance (5). Therefore, surveillance designers often use the so-called *enhancements* to mitigate the risk of non-compliance by promoting the desired behavior in different ways. For passive surveillance activities, this could, [for e.g., be in the form of financial rewards for notifications, training to increase awareness and recognition of clinical signs, awareness campaigns to improve recognition of disease and awareness of reporting obligations/procedures, payment of compensation for mitigation measures, provision of alternative routes of reporting such as a phone hotline or notification by SMS, subsidized testing cost, or some other form of benefit, such as farmers receiving advice in return (www.fp7-risksur.eu/terminology/faq)]. In a voluntary control program, similar types of design features have been used to promote the affiliation to and uptake of control strategies, for example by peer influence (6). These design features fall into the definition of nudges, but have so far not been assessed against the nudge theory nor scientifically validated or justified as to whether they are effective. Furthermore, to our understanding, such operational features often form part of professionals' individual experience with implementation of disease control and tend not be reported in the scientific literature and official documents, making it difficult to identify them by systematic literature reviews.

Several theoretical frameworks exist in nudging literature, and they capture somewhat different aspects related to behavioral influence. Examples are: the Nuffield ladder of intervention (7), which describes the relationship between the strength of an intervention and freedom of choice; MINDSPACE, developed by the UK Institute for Government, which provides a checklist of psychological approaches to apply when seeking behavioral change (8); and the EAST framework, which focuses on design elements related to the successful uptake of interventions (9). Additionally, Ly et al. (10) have presented a generic framework for categorizing nudges, irrespective of how they are implemented.

This study was conducted as part of the ANIHWA-funded project SANTERO (risk-based Surveillance for ANimal healTh in EuROpe, running 2016–2017). The project as a whole aimed to promote further development of risk-based surveillance methods and provide support for their dissemination and integration in existing surveillance routines (santero.fp7-risksur.eu). The objective of this study was to carry out an inventory of animal health surveillance and disease control strategies and designs that have been implemented in the past with the intention of improving acceptance of, and compliance with, a particular surveillance and/or control activity. More precisely, the study aimed to: (1) identify design features in current and past animal health surveillance and control programs that aim to influence behavior and (2) classify and describe them according to the nudge theory. The study did not aim to evaluate the efficacy of the identified strategies.

## Materials and Methods

In this study, as described in detail below, experts in seven European countries were interviewed to collect information on behavioral influence strategies used in the surveillance and control of infectious agents in animals. In brief, the identified strategies were first graded according to the level of intrusiveness (Nuffield ladder). Thereafter, strategies graded to have a lower level of intrusiveness, i.e., the potential nudges, were classified according to attributes in the EAST framework, i.e. *Easy, Attractive, Social*, and *Timely*. As a third step, strategies including three or more of the EAST elements were described according to the MINDSPACE framework to pinpoint which psychological mechanisms the strategy was likely to activate to enhance performance of the desired behavior.

### Study Participants

Professionals with practical experience in designing and implementing surveillance and control programs, from seven countries in Europe [Sweden (SE), the Netherlands (NL), Switzerland (CH), Norway (NO), Denmark (DK), Ireland (IE), and Northern Ireland (NI)], were recruited *via* the network of surveillance experts involved in the SANTERO Consortium. The selection of participants was purposive and followed a snowball sampling approach. SANTERO partners were asked to provide initial suggestions of suitable experts from their countries. These were subsequently contacted, and if agreeing to participate, they were further asked to advise on other professionals to interview. In all, 37 experts were contacted and 24 accepted participation.

### Data Collection

Data were collected by interviewing the experts. An interview protocol was developed with the aim of capturing strategies meant to facilitate a desired behavior in the implementation of animal health surveillance and disease control activities. Desired behaviors included, e.g., enrollment in a control program, engagement in surveillance activities, compliance with the rules and regulations of animal health programs, or adoption of certain biosecurity practices. Examples of strategies were provided in the protocol to aid understanding of the scope of data collection. Collection of other relevant contextual information was also included in the protocol, such as information about the respondent's role and past or current involvement in animal health programs. The protocol was shared with the respondents after they had agreed to participate, but before the interview took place (Supplementary File 1). The interviews were performed in English and were conducted in person, by telephone, or by online meeting applications in April and May 2017. They lasted between 45 and 60 min and were recorded to facilitate recollection.

The interviewees came from different fields (livestock, wildlife, and aquaculture), and the interview openly asked about strategies applied within that context and in relation to specific target groups, primarily farmers, hunters, and the general public. In light of the diversity in the respondents' experiences, the scope was widened to include also more intrusive (non-nudge) strategies from the field of animal health management, including also strategies for implementing statutory activities. In addition, although livestock producers, hunters, and the general public were the main stakeholders of interest, strategies directed toward professionals with other roles in relation to animal health were also considered.

### Data Editing

Extracts of interest were transcribed, anonymized, and checked for accuracy. Transcripts and notes were then screened with special focus on information about strategies with potential behavioral influence, aiming to identify the desired behavior. For each strategy, the following variables were documented: desired behavior, implementer, stakeholder to influence, and type of activity (i.e., surveillance activity, control scheme, general biosecurity program, biosecurity measure, or general animal health management), whether the strategy was voluntary or compulsory or was implemented during the voluntary or compulsory phase of a control program, animal species, pathogen/s, and country of implementation. The (perceived) efficiency of the strategy was also noted, in the case this was reported. The data were entered into a spreadsheet (Microsoft Excel 2016).

The variables extracted from the data were categorized to facilitate analysis. The variable desired behavior was categorized into: (1) “enroll and/or engage in a control program,” such as becoming aware of an animal health activity or enrolling into a biosecurity or control program, or actively proceeding to a higher level in such programs; (2) “engage in surveillance,” including, e.g., submitting samples or reporting dead animals; (3) “comply with surveillance and control activities,” i.e., following stated rules and regulations, irrespective of whether they are compulsory or not and irrespective of the legal basis [e.g., culling of persistently infected (PI) animals and trade requirements within a control program]; (4) “adopt biosecurity practices,” such as performing a proper biosecurity behavior on the farm or in conjunction with transport; and (5) “accept surveillance,” including, e.g., strategies to enhance sampling once a disease has been eradicated or within a control program, aiming at the long-term acceptance of the activity. The type of activity was categorized as: (1) control program; (2) surveillance; (3) biosecurity program; (4) general biosecurity practices; and (5) general animal health management.

**Figure 1:**
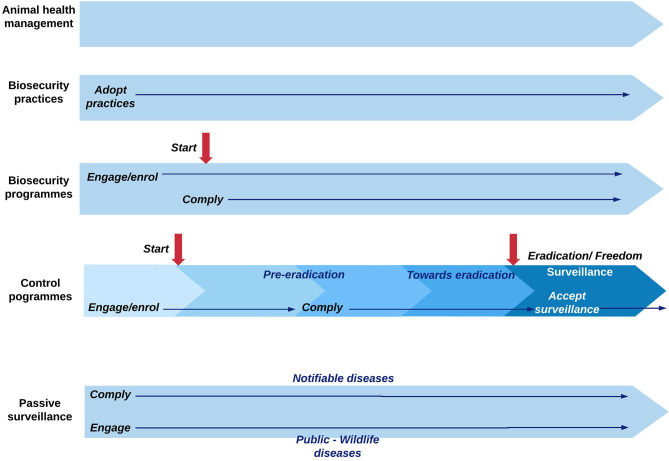
Overview of the timeline of animal health activities in relation to the application of strategies to achieve a behavior of interest.

Implementers were categorized as: (1) animal health (AH) providers; (2) industry, meaning the policy level rather than individual companies; and (3) authorities. Both AH providers and industry are private implementers, but in this context, the difference is that AH providers have, as the main task, to address animal health issues at a more individual or herd level, whereas industry strategies act at a higher level, in the form of decisions or norms covering all the farmers. Stakeholder to influence was categorized into: (1) producers; (2) hunters; (3) general public; (4) animal health providers; (5) industry stakeholders; (6) authorities; and (7) other groups, e.g., farm and abattoir workers, transporters, or traders. The phase of the activity was categorized as: (1) voluntary, when it was voluntary to perform the desired behavior or when the strategy was implemented during the voluntary phase of a program; (2) compulsory, when it was compulsory to perform the desired behavior or when the strategy was implemented during the compulsory phase of a program; and (3) voluntary and compulsory, when the strategy was implemented throughout a voluntary and a compulsory phase of a control program. Figure 1 provides an outline of the relationships between the categories of the variables “desired behavior,” “type of activity,” and “phase of program” (in relation to a timeline).

### Data Analysis

#### Application of the Framework

We described the strategies according to three theoretical frameworks, namely, the Nuffield ladder of intervention, EAST, and MINDSPACE. This work was done by all three authors jointly, using consensus decisions.

Firstly, the different strategies were graded according to the Nuffield ladder of intervention (7), which reflects the degree of intrusiveness of a policy, ranging from the least intrusive to the strongest: “Provide information,” “Enable choice,” “Guide choice through change of default,” “Guide choice through incentives,” “Guide choice through disincentives,” “Restrict choice,” and “Eliminate choice.” Potential nudges would, by definition, be found at the lower, non-restrictive, levels. Consequently, strategies consisting of delivery of information or education on animal health were put in the lowest level of the Nuffield ladder. At the next level, which is the enabling of individual choice, we included strategies such as facilitating reporting of disease or enrollment into a program. At the third level, strategies guiding choice by changing the default policy to achieve a desired behavior were included, whereas strategies guiding individual choices by using incentives or disincentives, such as compensations or fines, fell into the fourth level. At the last two levels, strategies restricting or eliminating choices were included, for example regulatory approaches such as imposing quotas or import limits.

For the purposes of this paper, where the aim was to focus on strategies with potential nudging effect, the subsequent analysis was primarily focused on strategies that fell within the lower levels of the Nuffield ladder, i.e., “Provide information,” “Enable choice,” “Guide choice through a change in the default policy,” and “Guide choice through incentives or disincentives.” These strategies were contrasted against the EAST framework (9), which stands for *Easy, Attractive, Social*, and *Timely*. These are attributes that characterize approaches with high potential to engage and activate people, and thereby triggering a desired behavior. *Easy* focuses on how the strategy makes it easier for the individual to perform a desired behavior, e.g., by framing information in a simple manner, or by using default options to reduce effort, or by making it easy to carry out the desired action, such as submitting samples or signing up for a program. *Attractive* means that the strategy should be designed so that it is desirable to perform the behavior. This can be by attracting attention, e.g., by personalizing the information or using social or individual reward systems that may provide a positive self-image. For example, biosecurity programs that involve scores and tests, or public availability of herd status on the web, or by a sign on the entrance door of the barn. In addition, strategies have a stronger behavioral influence when they take place in a *Social* context. This could be achieved by setting social norms and showing what others do, e.g., referring to how many other farmers have joined a program or have achieved a disease-free status in a control program. Commitments also make strategies social, such as publicly signing up for an animal health program. Finally, strategies are more likely to be successful if applied in a *Timely* manner. If individuals are approached when they are more receptive, they will be more likely to carry out the desired behavior. Sending reminders to farmers to cull persistently infected animals before opportunities to receive subsidies for doing so expire is an example of such timeliness.

For each strategy, one or several inherent attributes of the EAST framework were identified by the authors. To capture strategies that, at least theoretically, had a high likelihood of being successful, we focused on those that incorporated three or more of the EAST attributes in the subsequent work, when describing the strategies in depth by means of the MINDSPACE framework. This was done to obtain insight and to illustrate the potential psychological mechanisms involved when applying the strategy.

MINDSPACE provides a perspective that is complementary to EAST. It includes nine different psychological mechanisms by which behavior can be influenced (8). These are denoted *Messenger, Incentives, Norms, Defaults, Salience, Priming, Affect, Commitments*, and *Ego*. Briefly, the *Messenger* mechanism is based on how we as humans respond to information from different sources and has to do with who we find trustworthy and credible. In the animal health context, for example, a farmer might be more likely to engage when a respected colleague speaks about the value of enrolling into a control program than if the same message is delivered by another professional. *Incentives* act upon mental shortcuts, so-called *system 1 thinking* (11), and are geared toward avoiding losses. The effect of incentives varies according to type, magnitude, and timing (8). Examples include subsidies and payments to undertake diagnostic investigations for certain infections in the case of selected clinical signs. *Norms* are behavioral expectations or rules within a group of people and reflect the fact that information about how others behave can influence an individual's behavior. Hence, providing information to farmers on what others do, and/or capitalizing on relationships with other individuals in associations or networks to implement strategies, could be effective. *Defaults* have an effect when strategies are designed to make the wanted option easier to choose, thus facilitating options that lead to a desired behavior. An example is the use of active opt-out, which can be applied when seeking permission to use clinical samples for secondary purposes (such as surveillance or research), by asking animal owners to provide their answer if they want to opt out. The *Salience* mechanism triggers our inclination to turn attention to important things that we can relate to; these are likely to be novel, accessible, and simple (8). Losses are also, in general, more salient to us than gains. *Priming* has the effect of increasing the likelihood of performing a desired behavior after exposure to certain stimuli, even though not consciously processed. One example is when information about a control program is communicated in positive terms some time before potential program participants are approached for recruitment. *Affect* is based on the power of generating emotions through words, images, or events. According to this mechanism, biosecurity messages are more likely to be adopted if they are framed in dramatic terms. Some strategies promote *Commitment* of farmers by using contracts or by making their herd health status public since individuals try to be consistent with public promises and reciprocal acts. Finally, the *Ego* mechanism comes into play when strategies prompt a positive self-image, making individuals feel better about themselves. This could be producers wanting to advance in control programs or hunters participating in the surveillance of wildlife diseases with public health impact to compensate for an otherwise potentially negative public image. Unlike EAST, MINDSPACE was not used to filter for potentially more effective and successful strategies but rather to provide in-depth behavioral insights to strategies identified as potentially effective by EAST.

**Figure 2:**
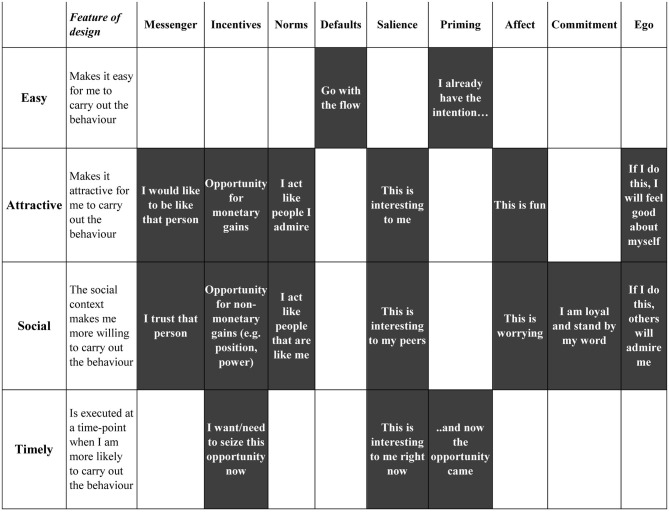
Interpretation of the relationships between EAST (Easy, Attractive, Social, and Timely) attributes and MINDSPACE (Messenger, Incentive, Norms, Default, Salience, Priming, Affect, Commitment, and Ego) psychological mechanisms in animal health interventions.

A matrix was developed by the authors, linking the interpretation of EAST attributes to MINDSPACE mechanisms (Figure 2) by exploring the underlying type of thinking. This matrix was used to provide guidance for the analysis and to validate the assessment of each strategy, supporting a systematic process during the analyses.

#### Statistical Analysis

Descriptive statistical results were produced from the variables extracted from the interviews. Associations between the type of activity, desired behavior, and implementer were tested for. In addition, associations between individuals and the cumulative number of EAST attributes vs. activity and implementer were checked. Contingency tables were generated, and homogeneity of the distributions were examined with Fischer's exact test, noting all combinations with a *p* < 0.1, liberally chosen considering the limited sample size and indicative nature of this study. For these analyses, Stata version 13 (Stata Statistical Software: Release 13, StatCorp LLP) was used.

## Results

In this section, we initially provide descriptive statistics of the collected strategies, i.e., country, disease, type of activity, implementer, and behavior to influence. Thereafter, we present the results of the application of the three study frameworks following a stepwise approach: firstly, the strength of the intervention by means of the Nuffield ladder; secondly, the attributes of the strategies based on the EAST framework including strategies in steps 1–5 in the Nuffield ladder; and, finally, the potential psychological mechanisms involved using the MINDSPACE framework, including strategies with design compatible with three or four of the EAST attributes.

### Descriptive Statistics

A total of 120 strategies were described in the interviews by 24 experts from seven countries. Between 11 and 22 strategies were reported per country, the most common context being control programs for endemic infections in cattle (*n* = 63), followed by surveillance activities for notifiable diseases (*n* = 31). Some general characteristics of the data are summarized in Table 1.

**Table 1:** Overview of the context within which strategies aimed at influencing behavior have been implemented in animal health activities in seven European countries.

**Country**	**No. of interviewees**	**No. of activities**	**No. of strategies**	**Species involved**	**Disease or pathogen**
Denmark	4	9	22	Cattle, mixed farm animals	Johne's disease, *Salmonella* Dublin, general AH, Aujeszky, Brucellosis, ASF, swine influenza, AMR, emerging diseases
Ireland	3	2	15	Cattle	BVD, general AH
Netherlands	4	10	17	Cattle, pigs, poultry, aquaculture, mixed	Notifiable diseases^a^, endemic diseases, aquatic bacterial zoonoses
Northern Ireland	1	3	13	Cattle, mixed farm animals	BVD, Johne's disease, general AH
Norway	3	6	22	Aquaculture, cattle, pigs, poultry wildlife	General AH, ISA, CWD, MRSA, *Mycoplasma hyopneumoniae*, notifiable diseases, BVD, IBR, EBL, *Brucella abortus*, Scrapie
Sweden	5	6	20	Cattle, pigs, sheep, wildlife	General AH, BVD, *Salmonella* Dublin, Q fever, *Echinococcus multilocularis*, Visna Maedi
Switzerland	4	8	11	Cattle, equids, wild birds, bees,	Beetle bee, general AH, BVD, AMR, BSE, BT, notifiable diseases, AI
Total	24	26	120		

*AH, animal health; BVD, bovine viral diarrhea; AI, avian influenza; AMR, antimicrobial resistance; BSE, bovine spongiform encephalopathy; CWD, chronic wasting disease; ISA, infectious salmon anemia; MRSA, methicillin-resistant Staphylococcus aureus: ^a^Notifiable diseases: avian Influenza, Newcastle disease, African swine fever, classical swine fever, foot, and mouth disease, Schmallenberg, and equine herpes virus, among others*.

There was an association (*p* < 0.1) between the behavior of interest to influence (i.e., “desired behavior”) and the activity in question. The most frequent behaviors in control programs were enrollment into the programs and compliance with different activities, whereas engagement was the most frequent goal for the strategies used in surveillance activities (Table 2). More than half of the strategies (*n* = 75) were implemented by the private sector, i.e., by animal health services (*n* = 51) or the industry (*n* = 24), whereas authorities were responsible for the implementation of one third of the strategies (*n* = 41). Further, the media was mentioned two times, as an actor providing information about surveillance activities run by authorities concerning diseases of public health impact. There was an association between the type of activity and the implementer; authorities were mostly responsible for engagement or compliance with surveillance for notifiable diseases as compared to animal health services that mostly were responsible for enrollment and engagement into control programs.

**Table 2:** Number of strategies per type of activity and per implementer, in relation to the desired behavior they intend to promote.

	**Categories**	***n***	**Adopt practices**	**Enroll and engage**	**Engage in an activity**	**Comply with an activity or program**	**Accept (surveillance)**
Activity	Animal health management	13	1	3	8	1	0
	Biosecurity training	9	9	0	0	0	0
	Biosecurity program	4	0	2	0	2	0
	Control program	63	7	25	0	26	5
	Surveillance	31	0	0	26	5	0
	Total	120	17	30	35	33	5
Implementer	Animal health providers	51	10	19	6	16	0
	Authorities	41	1	4	24	10	2
	Industry	24	6	7	2	6	3
	Authority and industry	2	0	0	2	0	0
	Media	2	0	0	1	1	0
	Total	120	17	30	34	33	5

*The aim of each strategy is to influence a certain desired behavior in a stakeholder*.

### Application of the Nuffield Ladder

Table 3 shows the strategies according to the level of intrusiveness of the intervention, i.e., according to the Nuffield ladder. Most strategies fell into the lowest level of intrusiveness, i.e., provision of information (*n* = 40), and 91 of the 120 strategies were classified within levels 1–5 in the Nuffield ladder, i.e., potential nudges. Animal health providers and authorities mostly used interventions in the lower steps of the ladder, namely, provision of information or changing the default to enable or guide choices. Strategies at level 4 or 5 of the Nuffield ladder, i.e., using incentives and disincentives, were frequently used by the industry (*n* = 12), but also by animal health providers (*n* = 14) and authorities (*n* = 10). Of the eight strategies graded as restricting or eliminating choices for the stakeholder, i.e., the highest steps in the Nuffield ladder, five were implemented by the industry and one by industry and authority together. More than half of the strategies (40/75) applied in the voluntary phase of control programs were graded to be in the three lowest steps of the ladder. In contrast, incentives and disincentives were more common in strategies performed during the compulsory phase of control programs (15/42).

**Table 3:**
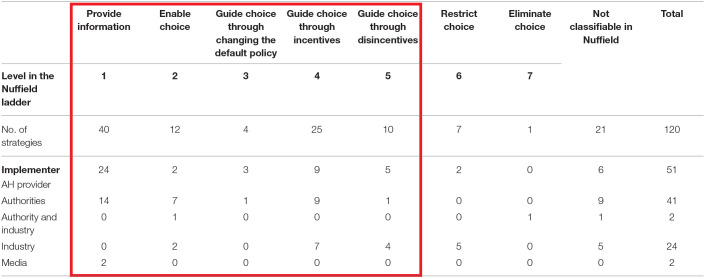
Overview of strategies based on the strength of intervention (the Nuffield ladder) and relation to the implementer.

*We focus on the strategies from levels 1 to 5*.

Twenty-one strategies were excluded from grading according to the Nuffield ladder because these strategies aimed to improve the efficiency of the system as a whole and did not target the behavior of individuals; thus, grading the intrusiveness was not relevant. These strategies were most frequently used in control programs (13/21), but also in animal health management activities (5/21) and, to a small extent, in surveillance activities (3/21). Some of these strategies (*n* = 5) aimed to enhance acceptance of surveillance activities within a program, after eradication or in the absence of disease.

### Application of the EAST Framework

**Figure 3:**
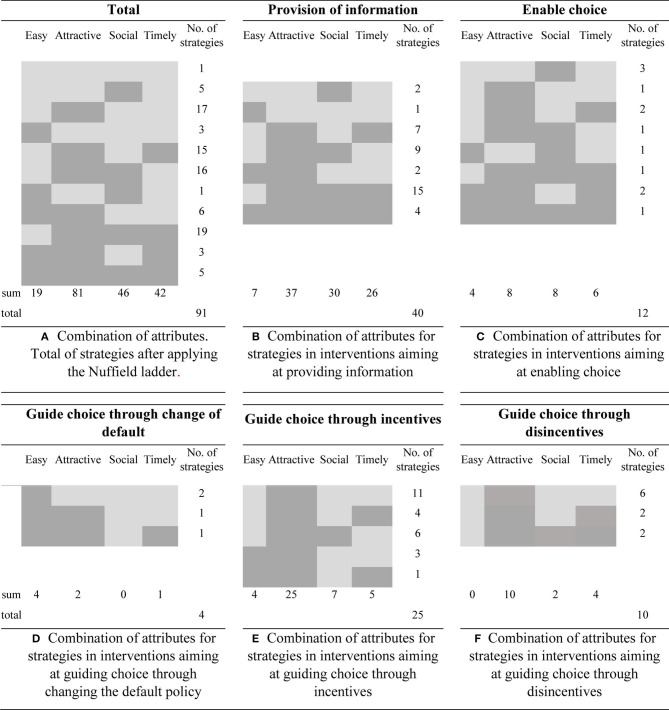
Combinations of attributes according to the EAST (Easy, Attractive, Social, and Timely) classification (dark gray) of strategies aimed at influencing behavior in the implementation of animal health activities. The results are shown as total and divided into the five lowest steps in the Nuffield ladder: total **(A)**; provision of information **(B)**; enable choice **(C)**; guide choice through change of default **(D)**; guide choice through incentives **(E)**; guide choice through disincentives **(F)**.

Figure 3 shows the patterns of attribute combinations obtained by applying the EAST framework to those strategies graded to be in the five lower levels of the Nuffield ladder, i.e., from information provision to the use of disincentives to guide choices. In all, 81 strategies included features that made it Attractive to perform a desired behavior; this was the most frequent attribute in all the activities. The least exploited attribute was Easy (19/91). There was an association (*p* < 0.1) between the type of activity where the strategy was used and the number of EAST attributes incorporated in the strategy, with surveillance activities and general biosecurity programs having a higher number of EAST attributes than did the control programs, biosecurity training activities, and animal health management activities. There was no association between the individual EAST attributes and the implementer, nor between the number of EAST attributes and the implementer. Of these 91 strategies in levels 1 to 5 in the Nuffield ladder, 22 incorporated three of the EAST attributes and five were classified to have all four attributes.

### Application of the MINDSPACE Framework

Table 4 lists the 27 strategies having three or four EAST attributes and also includes the MINDSPACE psychological mechanisms identified for those strategies. These are described in more detail below from section Strategies to Enroll or Engage a Target Population Into a Control Program to section Strategies to Promote Adoption of Biosecurity Practices. Strategies with <3 EAST attributes and strategies outside the Nuffield ladder are illustrated in Supplementary Files 1, 2, respectively, and interpreted in Supplementary File 3.

**Table 4:** Characteristics of the selected strategies showing design features compatible with three or more EAST attributes.

**n**	**Desired behavior**	**Country**	**Species**	**Activity**	**Phase**	**Disease/pathogen**	**Description**	**Implementer**	**Intervention ladder**					**E**	**A**	**S**	**T**	**M**	**I**	**N**	**D**	**S**	**P**	**A**	**C**	**E**
									**1**	**2**	**3**	**4**	**5**													
1	Enroll; Engage	SE	Cattle	Control prog	V	BVD	Lunch meetings organized by regional CVO	Authorities																		
2	Enroll; Engage	NI	Cattle	Control prog	V	BVD	Talks in agricultural shows. Social context and message framed.	AH provider																		
3	Enroll; Engage	NI	Cattle	Control prog	V	BVD	Farmers' testimonies on the impact of disease and benefits of a control program	AH provider																		
4	Enroll; Engage	IE	Cattle	Control prog	V	BVD	Talks in agricultural markets on program delivered by trusted sources	AH provider																		
5	Enroll; Engage	SE	Cattle	Control prog	V	BVD	Ambassadors, farmers with positive experiences to convince others	AH provider																		
6	Enroll; Engage	DK	Cattle	Control prog	V	*Salmonella* Dublin	Framed information (to convince, persuade stakeholders)	AH provider																		
7	Enroll; Engage	IE	Cattle	Control prog	V	BVD	Framed info in meetings and messenger (trusted source)	AH provider																		
8	Enroll; Engage	CH	Cattle	Control prog	V	BVD	Use of multipliers to deliver a framed message to stakeholder	Authorities																		
9	Enroll; Engage	NO	Pigs	Control prog	V	*M. hyopneumoniae*	Ambassadors, testimonies of other key farmers	AH provider																		
10	Engage	CH	Cattle	Surveillance	V	AMR	Use of multipliers to deliver a framed message to stakeholder	Authorities																		
11	Engage	CH	Wild birds	Surveillance	V	Avian influenza	Awareness campaign. Trust and engagement with wildlife guards	Authorities																		
12	Engage	CH	Bee	Surveillance	V	Beetle bee	*Apinella*. Participation; information; usefulness of system	Authorities																		
13	Engage	NL	Poultry	Surveillance	C	Avian influenza	Effect of outbreaks on farmer and community as salient event	Authorities																		
14	Engage	NL	General	Surveillance	C	Notif diseases	Ambassadors/testimonies on impact of disease	Authorities																		
15	Engage	NL	General	Surveillance	C	Notif diseases	Framed info: impact and consequences of outbreak, not being the source of infection to neighbor	Authorities																		
16	Engage	NO	Wildlife	Surveillance	V	CWD	Media acting as amplifiers of information. Raise awareness	Media																		
17	Engage	CH	Equids	Surveillance	V	Non-notif diseases; unspec. symptoms	*Equinella* system, incentives to report, usefulness of system	Authorities																		
18	Engage	NL	Poultry	Surveillance	C	Livestock diseases	Exclusion diagnostics as incentive to submit	Authorities																		
19	Engage	DK	Cattle	AH management	V	General AH	Framed information, trust on providers and authorities	AH provider																		
20	Engage	NL	General	AH management	V	Endemic diseases	Journals (with framed information) + use of newsletters	AH provider																		
21	Comply	NI	Cattle	Control prog	C	BVD	Disclose information to neighbor about persistently infected animals	AH provider																		
24	Comply	IE	Cattle	Control prog	C																					
22	Comply	DK	Cattle	Control prog	V	S Dublin	Public availability of farm status to encourage improvement, promote responsible behavior, avoid stigma	AH provider																		
23	Comply	IE	Cattle	Control prog	C	BVD	Vet involved in testing. Build on trust, commitment	AH provider																		
25	Comply	IE	Cattle	Control prog	C	BVD	Targeted advisory system for animal health, investigation of positive herds. SMS-based	AH provider																		
26	Comply	CH	Cattle	Surveillance	C	BSE	Media acting as amplifiers of message. Responsibility of vets to report	Media																		
27	Adopt BP	DK	General	Biosecurity	V	Biosecurity	Transport standards. Cleaning and disinfection—effortless process	AH provider																		

*In “activity”: Control prog, control program; AH mngmnt, animal health management. In “phase”: V, voluntary phase; C, compulsory phase. In “implementer”: AH provider, animal health provider. In “intervention ladder”: 1, provision of information; 2, enable choice; 3, guide choice through a change in default policy; 4, guide choice through the use of incentives; 5, guide choice through the use of disincentives. In “EAST”: E, easy; S, social; A, attractive; T, timely. In “MINDSPACE”: M, messenger; I, incentive; N, norms; D, default; S, salience; P, priming; A, affect; C, commitment; E, ego*.

#### Strategies to Enroll or Engage a Target Population Into a Control Program

Of the 27 strategies that captured three or four EAST attributes, nine were aimed at voluntarily *enrolling or engaging people in a control program* (Table 4). For most, emphasis was placed on the context in which information was delivered (social and timely) and by framing messages in an attractive way. For example, experts from IE and NI reported delivering talks at agricultural shows/markets using animal health service staff who run control programs (strategies 2 and 4 in Table 4). The aim was to attract farmers' attention using information about the benefits of schemes and costs of disease. These strategies are likely to incorporate at least four MINDSPACE mechanisms: Messenger, Norms, Salience, and Priming. Presenting positive information in a conducive social context builds on the idea that farmers become more receptive when later approached for enrollment. These strategies also capitalize on the potential social and normative effects of delivering information to a group sharing similar characteristics. In this context, the animal health services were described by interviewees as a positively regarded information provider. Also, among the strategies capturing four EAST attributes, there were farmers sharing testimonies (strategy 3 in Table 4) on the impact of diseases and positive experiences when enrolling in a control program, [for e.g., in conjunction with social events (markets and agricultural shows)]. This can potentially prime attendees for later enrollment in a program. In general, farmers acting as messengers by sharing their experiences with other farmers is a strategy used in almost all countries. This type of “ambassadorship” was described by some study participants (strategies 5, 8, and 9 in Table 4) as well-received by farmers, in particular if the farmer acting as a messenger had a good community image and strong position. Farmers also volunteered as pioneer ambassadors in the early stages of control/eradication schemes and actively tried to convince reluctant farmers to join. From a MINDSPACE perspective, mechanisms like Messenger, Norms, Salience, Affect, and Ego are likely to have played a role in these strategies.

Another example of a strategy with four EAST attributes was reported from SE: a strategy to promote enrollment in a control program (strategy 1 in Table 4) in which the Chief Veterinarian, regionally responsible for the implementation of the bovine viral diarrhea (BVD) eradication scheme on the isle of Gotland, invited dairy farmers to lunches, grouped according to probable BVD status (based on the results of a national bulk milk screening conducted before the scheme started). There, they received information about the control program and the disease, tailored to their specific BVD status. This strategy included effective targeting and personalization of information in a conducive social environment, putting farmers at comfort and ease. At the end of the lunch meeting, an enrollment list was circulated. In this case, most MINDSPACE mechanisms were actioned: a trustworthy Messenger; a message and context possibly involving Salience and Affect; and a social situation playing on Norms, Commitment, and Ego, i.e., by seeing their peers signing up, farmers were inclined to follow, and/or due to the sense of reciprocity, they wanted to maintain a good image. An additional feature was that the enrollment lists included all farmers that were invited, so attendees could see who had not attended. Reportedly, attending farmers later approached non-attending peers with the message, possibly Priming them to sign up later.

For some strategies, the stakeholders to be influenced were decision makers, not farmers. Provision of framed information in a social context to persuade and influence the engagement of decision makers in control programs was mentioned in IE and DK (strategies 6 and 7 in Table 4). In these examples, information on disease losses caused by BVD and Salmonellosis in cattle as well as the evidence of the costs and benefits of the programs for the farmers were presented to decision makers in the industry to obtain their support and engagement for the program. Salient information on the benefits of the program and potential losses and impact of the disease can be interesting to the audience and act as an Incentive mechanism. In IE, framing the information, focusing on disease losses and benefits of the control program and responsibility of the farmer, was combined with using a Messenger. In this case, animal health providers presented the information, and they were regarded as experienced and reliable sources of information, based on published scientific articles. This was described to be essential to generate trust and promote engagement.

#### Strategies to Engage a Target Population in Surveillance Activities

Eleven of the 27 strategies incorporating three or more EAST attributes promoted *engagement in surveillance activities*. In CH, media campaigns (strategy 11 in Table 4) were used to convey information about avian influenza (AI) surveillance and encourage the general public to report wild bird mortality events and to submit samples. The campaign used wildlife guards as trustworthy Messengers and also activated Norms, Salience, and Ego as these officers are highly regarded sources of clear and reliable information and have a large “social capital” (compared to authority employees or veterinarians). Another example, including two strategies (nos. 46 and 47 in Supplementary File 1), was the engagement of hunters in surveillance of *Echinococcus multilocularis* in SE. In this case, the authorities used the hunters' social networks to broadcast information and promote submission of fecal samples from foxes for national screening, emphasizing the importance of their contribution to society (Salience and Norms). A symbolic compensation was paid (Incentive), but not to individual hunters; instead, the fee went to the local hunters' clubs. This could positively affect engagement, potentially triggering hunters' sense of Commitment (to the club and to society) and a positive self-image (Ego). A positive image in society and justification of hunting as an activity could also have played a normative role, particularly as hunting may be controversial. With time, participation decreased. The authorities then tried to improve the attractiveness and timeliness of their information by using a mailing list to deliver updated key information through the hunters' network. The same network was used to transmit pre-hunting season reminders about the importance of passive surveillance and availability of free postmortem services for wildlife, information that is Salient to hunters, possibly Priming them to act when asked to send in samples for surveillance.

Some strategies used to engage people in surveillance activities were launched at time points when the target groups were more receptive, similar to the enrollment strategies for endemic disease control programs (strategies 10, 13–15, and 19 in Table 4). In general, novel or unexpected events with high impact (e.g., exotic disease outbreaks) were reported from all countries as an opportunity for engaging stakeholders in surveillance activities (strategy 13, Table 4) due to their Salient nature. Such events are also perceived as generating enhanced receptiveness for information on biosecurity and animal health management in general. This was seen across all types of animal production in this study. For example, during the onset of AI outbreaks in the NL, the “ambassador” strategy was used (strategy 14, Table 4), with farmers previously suffering the disease serving as credible Messengers to others within their network, reducing fear of economic and social consequences and enhancing willingness to report events.

Some strategies aimed to reduce fear of negative consequences from, e.g., authorities control measures or societal stigma. An example of this was a streamlined process for farmers in the NL so that they could submit samples to the diagnostic point without needing to notify authorities or practitioners until results were available. This would enhance submission of samples during higher and transient mortality or unspecific symptoms, making it Easy and Timely and reducing the potential stigma (strategy 18, Table 4). In Switzerland, early detection systems for diseases in different species have been designed to enable and promote voluntary participation of farms acting like sentinels. Examples of this are the *Apinella* and *Equinella* systems (strategies 12 and 18, Table 4). The former intended to recruit bee producers (12), whereas *Equinella* targeted veterinary practitioners (13).

In NL, information to engage farmers into animal health management and surveillance activities (strategy 20, Table 4) was provided using several different strategies that could potentially be addressing all the attributes. Journals and newsletters were used to provide information of interest or to share stories of other farmers at times when the target audience would be more susceptible, with the aim of increasing attention regarding different topics or providing a priming effect for the future.

#### Strategies to Improve Compliance With Activities

Six strategies aimed to improve *compliance with surveillance and control activities*. These had three EAST attributes (Attractive, Social, and Timely) and aimed to improve uptake of voluntary, but strongly recommended, activities within control programs during their compulsory phase or at higher levels of industry-run biosecurity programs. In the compulsory phase of the control programs, “Providing information” was often combined with stronger interventions, such as guiding choices through incentives or disincentives (Table 4). These strategies were often built on a coaching type of interaction between a veterinary practitioner or advisor and a farmer. In this setting, personalized information and advice can be provided, and planning and implementation can be participatory, to facilitate farmer engagement. One example came from the compulsory phase of the BVD scheme in IE (strategies 23–25, Table 4), where culling of PIs was strongly recommended, but yet a voluntary measure (no legal requirement). The “nudge” was a timely text message (SMS) to farmers with a reminder to remove persistently infected animals at key time points. However, the effectiveness of this strategy is built on a solid relationship between the farmers and veterinary practitioners, supported by a farmer-targeted advisory online IT system. The veterinary practitioners were involved in all testing and tagging procedures (strategy 23, Table 4) and could thereby provide encouraging advice to farmers in conjunction with such interactions. The involvement of the farmer was necessary as they reported when required visits had been conducted. Also, monitoring data were used to establish the most likely routes of infection and to tailor biosecurity messages. Consequently, provision of information becomes timely, and although from an IT system, its Messenger is trustworthy, Defaults are built into the supportive system, Salience is elicited, and farmer Commitment is encouraged (and required) throughout the program. Furthermore, linked to the system results on persistently infected animals retention, neighboring farms received a notification. Public availability of herd status or selected test results was used in some strategies to promote compliance during the control and eradication programs (strategy 22, Table 4). For instance, in DK, *Salmonella* Dublin herd status based on test results from all dairy herds in the country was publicly available from the start of the program. The information is Attractive for the farmers as it is interesting to know their own herds' status, but they can also check the status of neighboring herds and any changes over time. Potentially, this could create a Normative effect and thereby encourage the progression in the program to achieve a higher *S*. Dublin status.

#### Strategies to Promote Adoption of Biosecurity Practices

Of the strategies that aimed to encourage *adoption of biosecurity practices* in general, not linked to specific disease control programs, none addressed all four attributes of the EAST framework and only one was considered to address three attributes. This was the Danish policy for transport requirements with regard to cleaning and disinfection (strategy 27, Table 4), in which access to washing facilities in trade operations is designed to be effortless, as well as the payment, as transporters do not need to deal with it.

## Discussion

In this study, experts in seven countries were interviewed to capture strategies that have been used within this context to influence the behavior of a certain target group. A large number of strategies were described in the interviews, including not only typical nudges but also a variety of strategies to improve the performance of control programs and surveillance activities. We can confirm that strategies to influence behavior have been commonly used in the implementation of animal health surveillance and disease control activities in several European countries, and the examples collected in this study may serve as inspiration for colleagues when designing strategies in current and future control and surveillance programs.

The strategies described to us were applied by different implementers, authorities, as well as industry and animal health services. It was interesting to note that the more forceful strategies, i.e., graded as higher steps in the Nuffield ladder, were mostly implemented by the industry, while those implemented by animal health organizations and authorities normally were in the lower part of the Nuffield ladder (Table 3). This has logic, as requirements for buying products will be necessary for a farmer to comply with in order to stay in business. Authorities, on the other hand, can issue laws and regulations. These may seem forceful, but in the end, how well laws and regulations are complied with will likely depend on the same psychological mechanisms as voluntary measures.

In general, the design of the strategies described in this study has been based on professional empirical understanding of the requirements to achieve certain outcomes rather than the systematic use of methods from the behavioral sciences and psychology. Despite this, we found that almost three quarters (91/120) of the obtained strategies were likely to have triggered multiple psychological mechanisms that support conscious or intuitive actions (Table 4 and Supplementary File 1), of which 27 strategies included three or more desired design attributes (Table 4) according to the EAST framework. This is in line with the existing recommendations for successful influence on a behavior (8, 9, 14), which shows that intuition and a good understanding of the context may reach far. However, consideration of the psychological mechanisms to trigger may be helpful when designing strategies. Therefore, application of the frameworks used in this study may be useful as a supporting tool in future design of strategies in animal health and disease control programs.

Providing information was the most common form of strategy delivered in a wide range of contexts. Despite the differences in context, there were some common aspects in how content was framed and how context or environment was modified. For example, irrespective of the desired behavior, it was common to use a trustworthy Messenger to deliver information and to ensure that the context would trigger social mechanisms such as Norms, Commitment, and Ego.

The least used design attribute in the strategies was to make the desired behavior Easy to carry out, with only 19 of 91 strategies having this element. This indicates an opportunity for improvement in strategy design by, for example, considering defaults, reducing the “hassle factor” of carrying out a desired behavior, or simplifying messages [(9, 15)]. In particular, it could be important in maintaining compliance or acceptance of an intervention (16). In general, smartphone solutions are well-suited as nudging tools due to their potential timeliness and availability, making it easier for individuals to maintain a desired behavior (17). We obtained information on programs and surveillance activities implemented up to two decades ago when these technology solutions where not yet in place or as easily accessible as they are today. Therefore, it is likely that the group of strategies aiming to make behaviors easy by using technology already has increased. In the public sector, the so-called service design (18) is used with increasing frequency to engage citizens in the design of services. This approach, called service design, is particularly useful, and even necessary, to accurately capture a target group's experience in interacting with a company or public institution. Potentially, this approach could be one way by which strategies that support an Easy execution of the desired behavior could be designed.

In this study, we also identified the existence of positive *spin-off* effects. By this, we mean that some strategies ended up influencing a positive behavior in addition to the desired behavior. For instance, the use of a sign on the door in BVD-free herds in the Swedish control program was intended to promote biosecurity measures of visitors, but it also ended up creating a positive benchmarking effect on communities. Similarly, within the Danish *Salmonella* control program in cattle, publicly available herd test results were initially used to promote biosecurity and to contain the disease, but were also used as incentives for farmers with improving test results to take part in the program and actively move toward control and eradication.

This study did not include any evaluation of the efficacy of the different strategies. Still, some unsuccessful examples were shared with us during the interviews. For example, early over-communication of the public health risks of *E. multilocularis first* triggered a sense of commitment and societal responsibility in Swedish hunters, but this turned into disengagement in surveillance when they realized that the authorities did not take any measures once the infection was introduced. In the NL, the use of inappropriate Messengers (authority representatives informing poultry farmers about surveillance without having an understanding of the production system) resulted in farmers' disengagement due to lack of trust, with a subsequent negative impact on the reporting of disease events (data not shown). This shows that it is important to be aware that some MINDSPACE mechanisms, such as Salience and Affect, have less predictable outcomes as they depend on individual emotional associations that will influence the decision making (19). Also, behavioral influence strategies can sometimes backfire (16, 20) if perceived as condescending or activating psychological mechanisms of trust and commitment without solid justification.

There has been little implementation research related to behavioral influence strategies in animal health surveillance and disease control, and evaluation of the effect of the different strategies was not part of this study either. One study developed and applied an agent-based model to assess the effects of different strategies on the uptake of livestock vaccination for Blue Tongue in the Netherlands. Their model showed that, at the start of a livestock disease epidemic, specific scheme designs improved vaccine uptake by farmers in comparison to interventions with no improved design, further capturing interactions between strategies (21). Even though the evaluation of strategies would be desirable, it provides challenges as interventions often appear in combinations. The difficulties in assessing the effectiveness of individual behavioral interventions have been described in the public health sector (22, 23) and guidelines to conduct evaluation of complex interventions have been proposed (24, 25). One example of an ongoing work in the animal sector was mentioned during the interviews: a study in Danish farms where the effect of different nudges is studied to see whether they result in improved biosecurity behavior and reduced work-related injuries (SEGES, personal communication). Future studies to evaluate the effect of different interventions would be an interesting field of research and could also form an important basis for future decisions on the design of surveillance and control programs in the animal sector.

## Data Availability Statement

All datasets generated for this study are included in the article/Supplementary Material.

## Author Contributions

AL and MG conceived and designed the study. MG conducted the interviews, initial data screening, and editing. MG, EÅ, and AL conducted the analysis, wrote, and revised the manuscript. All authors contributed to the article and approved the submitted version.

## Conflict of Interest

The authors declare that the research was conducted in the absence of any commercial or financial relationships that could be construed as a potential conflict of interest.
