# Supplementary material for: Nudging in Animal Disease Control and Surveillance: A Qualitative Approach to Identify Strategies Used to Improve Compliance With Animal Health Policies
Source: Front Vet Sci. 2020 Aug 5;7:383. doi: 10.3389/fvets.2020.00383 (PMC7419428; doi:10.3389/fvets.2020.00383)
Supplement: Supplementary file 1 [file Table_1.DOCX]

**Supplementary File 1**. Characteristics of strategies 28 to 99. Considered to aim to address the individual primarily, but presenting less than three EAST attributes.

| **n** | **Desired behaviour** | **Country** | **Species** | **Activity** | **Phase** | **Disease/ pathogen** | **Description** | **Imple-**  **menter** | | **Intervention ladder** | | | | | | | | | | | | | | **E** | | **A** | | **S** | | **T** | | **M** | | **I** | | **N** | | **D** | | **S** | | **P** | | **A** | | **C** | | **E** | |  |
| --- | --- | --- | --- | --- | --- | --- | --- | --- | --- | --- | --- | --- | --- | --- | --- | --- | --- | --- | --- | --- | --- | --- | --- | --- | --- | --- | --- | --- | --- | --- | --- | --- | --- | --- | --- | --- | --- | --- | --- | --- | --- | --- | --- | --- | --- | --- | --- | --- | --- | --- |
|  |  |  |  |  |  |  |  |  |  | **1** | | **2** | | **3** | | **4** | | **5** | | **6** | | **7** | |  |  |  |  |  |  |  |  |  |  |  |  |  |  |  |  |  |  |  |  |  |  |  |  |  |  |  |
| 28 | Enrol; Engage | SE | Cattle | Control prog | V | BVD | Provision of information on National bulk milk screening pre scheme for free. | AH provider | |  | |  | |  | |  | |  | |  | |  | |  | |  | |  | |  | |  | |  | |  | |  | |  | |  | |  | |  | |  | |  |
| 29 | Enrol; Engage | IE | Cattle | Control prog | V | BVD | Multi-stakeholder meetings by county, with farmers organisations, to provide info EU situation options. (authority trust) - show what others do/norms. | AH provider | |  | |  | |  | |  | |  | |  | |  | |  | |  | |  | |  | |  | |  | |  | |  | |  | |  | |  | |  | |  | |  |
| 30 | Enrol; Engage | CH | Cattle | Control prog | V | BVD | Meetings with farmers to engage, those convinced using multipliers to tell their stories and experiences with the disease - pre BVD program implementation. | Authorities | |  | |  | |  | |  | |  | |  | |  | |  | |  | |  | |  | |  | |  | |  | |  | |  | |  | |  | |  | |  | |  |
| 31 | Enrol; Engage | DK | Cattle | Control prog | V | Johne's disease | Information on economic impact, losses of the disease. | AH provider | |  | |  | |  | |  | |  | |  | |  | |  | |  | |  | |  | |  | |  | |  | |  | |  | |  | |  | |  | |  | |  |
| 32 | Enrol; Engage | NI | Cattle | Control prog | V | BVD | Information on economic impact, losses of the disease, benefits control. | AH provider | |  | |  | |  | |  | |  | |  | |  | |  | |  | |  | |  | |  | |  | |  | |  | |  | |  | |  | |  | |  | |  |
| 33 | Enrol; Engage | NI | Cattle | Control prog | C | BVD | Education provision: literature, farmer press. Provision of information on compulsory measures, testing and restriction of movements between farms. | AH provider | |  | |  | |  | |  | |  | |  | |  | |  | |  | |  | |  | |  | |  | |  | |  | |  | |  | |  | |  | |  | |  |
| 34 | Enrol; Engage | DK | Cattle | Control prog | C | Salmonella dublin | Control to eradication period. Time given to join voluntarily to implement the plan before compulsory. | Industry | |  | |  | |  | |  | |  | |  | |  | |  | |  | |  | |  | |  | |  | |  | |  | |  | |  | |  | |  | |  | |  |
| 35 | Enrol; Engage | IE | Cattle | AH management | V | General AH | Existence of multi-stakeholder group to enhance communication. (regular meetings) | AH provider | |  | |  | |  | |  | |  | |  | |  | |  | |  | |  | |  | |  | |  | |  | |  | |  | |  | |  | |  | |  | |  |
| 36 | Enrol; Engage | CH | Cattle | AH management | V | General AH | Generation of dialogue and relationships between stakeholders: industry and farmers. | Authorities | |  | |  | |  | |  | |  | |  | |  | |  | |  | |  | |  | |  | |  | |  | |  | |  | |  | |  | |  | |  | |  |
| 37 | Enrol; Engage | SE | Cattle; Pigs | Biosecurity program | V;C | S dublin; Salmonella spp; AH management | Economic incentive: joining provides coverage/insurance/partial compensation if outbreak occurs or Salmonella is detected. | AH provider | |  | |  | |  | |  | |  | |  | |  | |  | |  | |  | |  | |  | |  | |  | |  | |  | |  | |  | |  | |  | |  |
| 38 | Enrol; Engage | SE | Cattle | Biosecurity program | V | AH Management | User friendly system, videos of farmers and their experiences, interactive information, animations. | AH provider | |  | |  | |  | |  | |  | |  | |  | |  | |  | |  | |  | |  | |  | |  | |  | |  | |  | |  | |  | |  | |  |
| 39 | Enrol; Engage | SE | Cattle | Control prog | V | BVD | Subsidies for sampling. Reduced costs for farmers. | Industry | |  | |  | |  | |  | |  | |  | |  | |  | |  | |  | |  | |  | |  | |  | |  | |  | |  | |  | |  | |  | |  |
| 40 | Enrol; Engage | SE | Cattle | Control prog | V | BVD | BVD free status gave an added value to calves creating a market effect. | Industry | |  | |  | |  | |  | |  | |  | |  | |  | |  | |  | |  | |  | |  | |  | |  | |  | |  | |  | |  | |  | |  |
| 41 | Enrol; Engage | NL | Cattle | Control prog | V | BVD; IBR | Farmers only pay a % of the full cost of the schemes. | Industry | |  | |  | |  | |  | |  | |  | |  | |  | |  | |  | |  | |  | |  | |  | |  | |  | |  | |  | |  | |  | |  |
| 42 | Enrol; Engage | NL | Cattle | Control prog | V | BVD; IBR | Quality programs based on points system: certain behaviours add or deduct points, e.g. not being part of IBR, BVD schemes. | Industry | |  | |  | |  | |  | |  | |  | |  | |  | |  | |  | |  | |  | |  | |  | |  | |  | |  | |  | |  | |  | |  |
| 43 | Engage | NL | Aquaculture | Surveillance | V | Bacterial diseases; zoonotic hazards | **Journals/literature/Lectures** in aquaculture society for stakeholders cohesion and discussion, involvement. **Workshops** for workers on zoonosis, network with hospitals. **Courses** to vets. Information on dissemination of diseases, hygiene practice. | Authorities |  | |  | |  | |  | |  | |  | |  | |  | |  | |  | |  | |  | |  | |  | |  | |  | |  | |  | |  | |  | |  |  |
| 44 | Engage | NO | Pigs | AH management | C | General AH | Information from industry and authorities to producers on legislation and biosecurity, tailored and personalised based production system needs. | Authorities |  | |  | |  | |  | |  | |  | |  | |  | |  | |  | |  | |  | |  | |  | |  | |  | |  | |  | |  | |  | |  |  |
| 45 | Engage | NL | General | AH management | V | Endemic diseases | Service for endemic diseases/ Helpdesk 5 days a week, 9 to 5pm. | AH provider |  | |  | |  | |  | |  | |  | |  | |  | |  | |  | |  | |  | |  | |  | |  | |  | |  | |  | |  | |  | |  |  |
| 46 | Engage | SE | Wildlife | Surveillance | V | E multilocularis | **Information provision**. Faming message. Education: sample collection. Info on: contribution to society and public health. | Authorities |  | |  | |  | |  | |  | |  | |  | |  | |  | |  | |  | |  | |  | |  | |  | |  | |  | |  | |  | |  | |  |  |
| 47 | Engage | SE | Wildlife | Surveillance | V | E multilocularis | Reminders of the strategy, using mailing list and quarterly report. | Authorities |  | |  | |  | |  | |  | |  | |  | |  | |  | |  | |  | |  | |  | |  | |  | |  | |  | |  | |  | |  | |  |  |
| 48 | Engage | CH | Cattle | Surveillance | V | Blue Tongue | Producers-**participatory strategy**: sentinel farms, involved in elaboration of a clinical report and sample collection for monitoring of midges and some payment. | Authorities |  | |  | |  | |  | |  | |  | |  | |  | |  | |  | |  | |  | |  | |  | |  | |  | |  | |  | |  | |  | |  |  |
| 49 | Engage | DK | Wild boar | Surveillance | V | Aujeszky; Brucellosis; ASF | Building of **network** of hunters, working closely to remove practical barriers. | Authorities |  | |  | |  | |  | |  | |  | |  | |  | |  | |  | |  | |  | |  | |  | |  | |  | |  | |  | |  | |  | |  |  |
| 50 | Engage | NO | Pigs | Surveillance | C | Swine viruses | Promotion of **network**/strong relationship between authorities, industry and advisory bodies. | Authorities; Industry |  | |  | |  | |  | |  | |  | |  | |  | |  | |  | |  | |  | |  | |  | |  | |  | |  | |  | |  | |  | |  |  |
| 51 | Engage | DK | Wild birds | Surveillance | V | Avian influenza | **System facilitating reporting**: accessibility, transparency: phone, website, app for birdwatchers. | Authorities |  | |  | |  | |  | |  | |  | |  | |  | |  | |  | |  | |  | |  | |  | |  | |  | |  | |  | |  | |  | |  |  |
| 52 | Engage | SE | Sheep | Surveillance | V | Maedi Visna | Sampling for Maedi Visna same time of vaccination campaign for Blue Tongue. | AH provider |  | |  | |  | |  | |  | |  | |  | |  | |  | |  | |  | |  | |  | |  | |  | |  | |  | |  | |  | |  | |  |  |
| 53 | Engage | SE | Cattle | Surveillance | V | Q Fever | **Opt-out system**: made difficult to refuse the use of samples for other purposes. Opt-out forms to be sent by post. | Authorities |  | |  | |  | |  | |  | |  | |  | |  | |  | |  | |  | |  | |  | |  | |  | |  | |  | |  | |  | |  | |  |  |
| 54 | Engage | NL | General | Surveillance | C | Notifiable diseases | Development of more rapid and accurate test to **shorten period of uncertainty** - between submission of samples and results. | Authorities |  | |  | |  | |  | |  | |  | |  | |  | |  | |  | |  | |  | |  | |  | |  | |  | |  | |  | |  | |  | |  |  |
| 55 | Engage | NO | Wildlife | Surveillance | V | CWD | Rapid processing of the samples in 1-2 days and access to information on their submissions. Important for management of meat. **Shorten period of uncertainty.** | Authorities |  | |  | |  | |  | |  | |  | |  | |  | |  | |  | |  | |  | |  | |  | |  | |  | |  | |  | |  | |  | |  |  |
| 56 | Engage | NE | General | AH management | V | Endemic diseases | **System feature** to hide result for some time. | Industry |  | |  | |  | |  | |  | |  | |  | |  | |  | |  | |  | |  | |  | |  | |  | |  | |  | |  | |  | |  | |  |  |
| 57 | Engage | DK | Wild boar | Surveillance | V | Aujeszky; Brucellosis; ASF | **Provide results** on Trichinella (of **interest for farmers**) = time testing for other exotic diseases of interest. | Authorities |  | |  | |  | |  | |  | |  | |  | |  | |  | |  | |  | |  | |  | |  | |  | |  | |  | |  | |  | |  | |  |  |
| 58 | Engage | DK | Cattle | Surveillance | V | AMR to mastitis | To incentivise sample submission, more in depth analysis/info and pathogen **typing for** **free.** | Authorities |  | |  | |  | |  | |  | |  | |  | |  | |  | |  | |  | |  | |  | |  | |  | |  | |  | |  | |  | |  | |  |  |
| 59 | Engage | DK | Pigs | Surveillance | V | Swine flu viruses | When samples are submitted, **extra info is provided**: regarding specific vaccine needed, genotyping and sequencing **for free**. | Authorities |  | |  | |  | |  | |  | |  | |  | |  | |  | |  | |  | |  | |  | |  | |  | |  | |  | |  | |  | |  | |  |  |
| 60 | Engage | NL | Aquaculture | Surveillance | V | Vibrio | **Free diagnosis report** (information) on sampling on the farm: take samples and questionnaire. | Authorities |  | |  | |  | |  | |  | |  | |  | |  | |  | |  | |  | |  | |  | |  | |  | |  | |  | |  | |  | |  | |  |  |
| 61 | Engage | NO | Cattle | Surveillance | C | *Brucella abortus* | **Package** with Toxoplasma to get farmers more interested and committed to notify and send samples if two late abortions. | Authorities |  | |  | |  | |  | |  | |  | |  | |  | |  | |  | |  | |  | |  | |  | |  | |  | |  | |  | |  | |  | |  |  |
| 62 | Engage | DK | Cattle | Surveillance | V | Emerging diseases | Early detection of emerging problems in calves - Provide pathological and histopathological **free** service. Mailing list to engage farmers, provide information on results and feedback. | Authorities |  | |  | |  | |  | |  | |  | |  | |  | |  | |  | |  | |  | |  | |  | |  | |  | |  | |  | |  | |  | |  |  |
| 63 | Comply | SE | Cattle | Control prog | V;C | BVD | **Sign** on the door stating "BVD-free herd". | AH provider |  | |  | |  | |  | |  | |  | |  | |  | |  | |  | |  | |  | |  | |  | |  | |  | |  | |  | |  | |  | |  |  |
| 64 | Comply | NI | Cattle | Control prog | V | BVD | Engagement with stakeholders local farmer representative groups and organisations. Communication, meetings. | AH provider |  | |  | |  | |  | |  | |  | |  | |  | |  | |  | |  | |  | |  | |  | |  | |  | |  | |  | |  | |  | |  |  |
| 65 | Comply | NO | Pigs | Control prog | C | MRSA | **Communication between stakeholders**: **meetings, symposium and workshops** for farmers to give update information on findings and guidelines on practices and epidemiology of the disease – during eradication program. | Authorities |  | |  | |  | |  | |  | |  | |  | |  | |  | |  | |  | |  | |  | |  | |  | |  | |  | |  | |  | |  | |  |  |
| 66 | Comply | IE | Cattle | Control prog | V | BVD | **Database system** and link to laboratory results that the producers can access. | AH provider |  | |  | |  | |  | |  | |  | |  | |  | |  | |  | |  | |  | |  | |  | |  | |  | |  | |  | |  | |  | |  |  |
| 67 | Comply | IE | Cattle | Control prog | C | BVD | TASAH: **features of the system** in terms of communication, links to the database and SMS systems: farmers are sent messages re outcome of results and reminders to test animals or remove animals. | AH provider |  | |  | |  | |  | |  | |  | |  | |  | |  | |  | |  | |  | |  | |  | |  | |  | |  | |  | |  | |  | |  |  |
| 68 | Comply | NO | Poultry | Control prog | C | Salmonella spp. | Slaughtering houses have been given access to the sample **submission system**, to demand those producers their samples beforehand. | Authorities |  | |  | |  | |  | |  | |  | |  | |  | |  | |  | |  | |  | |  | |  | |  | |  | |  | |  | |  | |  | |  |  |
| 69 | Comply | NO | Pigs | Surveillance | C | Notifiable diseases | To proof freedom of disease/ Different **newsletters with update of information** to authorities, to local authorities, industry and farmers - **tailoring the message and findings to targeted group** ( nucleus farms, multipliers, sow pool) - and taking into account the epidemiological differences and importance of each group in regards to infectious diseases. | Authorities |  | |  | |  | |  | |  | |  | |  | |  | |  | |  | |  | |  | |  | |  | |  | |  | |  | |  | |  | |  | |  |  |
| 70 | Comply | SE | Cattle | Biosecurity program | V | AH Management | Second stage - Veterinary visit to **provide tailored information.** | AH provider |  | |  | |  | |  | |  | |  | |  | |  | |  | |  | |  | |  | |  | |  | |  | |  | |  | |  | |  | |  | |  |  |
| 71 | Comply | SE | Cattle | Biosecurity program | V | AH Management | System that requires engagement by the farmer and workers and proactive behaviour. | AH provider |  | |  | |  | |  | |  | |  | |  | |  | |  | |  | |  | |  | |  | |  | |  | |  | |  | |  | |  | |  | |  |  |
| 72 | Comply | IE | Cattle | Control prog | C | BVD | **Payment** system to cover the cost of the disposal - stage procedure payment linked to the retention time. | AH provider |  | |  | |  | |  | |  | |  | |  | |  | |  | |  | |  | |  | |  | |  | |  | |  | |  | |  | |  | |  | |  |  |
| 73 | Comply | NI | Cattle | Control prog | C | BVD | Financial incentive. **Cost of replacing** the PI cow, not a compensation fee. PI removal was not compulsory, despite the scheme being in the compulsory stage. | AH provider |  | |  | |  | |  | |  | |  | |  | |  | |  | |  | |  | |  | |  | |  | |  | |  | |  | |  | |  | |  | |  |  |
| 74 | Comply | IE | Cattle | Control prog | C | BVD | Establishment of the **"negative herd" as a milestone.** | Industry |  | |  | |  | |  | |  | |  | |  | |  | |  | |  | |  | |  | |  | |  | |  | |  | |  | |  | |  | |  | |  |  |
| 75 | Comply | IE | Cattle | Control prog | C | BVD | When "negative herd status" is achieved, **discounts** for testing ( for monitoring) are applied by the lab. | Industry |  | |  | |  | |  | |  | |  | |  | |  | |  | |  | |  | |  | |  | |  | |  | |  | |  | |  | |  | |  | |  |  |
| 76 | Comply | IE | Cattle | Control prog | C | BVD | **Restriction** of the herd if PI animal has not been removed after 5 weeks. | AH provider |  | |  | |  | |  | |  | |  | |  | |  | |  | |  | |  | |  | |  | |  | |  | |  | |  | |  | |  | |  | |  |  |
| 77 | Comply | NI | Cattle | Control prog | C | BVD | **Full movement restriction** - to be lifted when PI is sacrificed. | AH provider |  | |  | |  | |  | |  | |  | |  | |  | |  | |  | |  | |  | |  | |  | |  | |  | |  | |  | |  | |  | |  |  |
| 78 | Comply | IE | Cattle | Control prog | C | BVD | Payment system linked to Beef Genomics and Data Programme (BGDP) – if participation and compliance. | Industry |  | |  | |  | |  | |  | |  | |  | |  | |  | |  | |  | |  | |  | |  | |  | |  | |  | |  | |  | |  | |  |  |
| 79 | Comply | NL | Poultry; Pigs | Control prog | C | Salmonella spp. | The status at the slaughterhouse varies according to the freedom of Salmonella - if not free a lower status is assigned ( less value) - **Quality control norms.** | Industry |  | |  | |  | |  | |  | |  | |  | |  | |  | |  | |  | |  | |  | |  | |  | |  | |  | |  | |  | |  | |  |  |
| 80 | Adopt BP | DK | Cattle | Biosecurity | V | General AH | **Game:** related to animal health management and biosecurity. | AH provider |  | |  | |  | |  | |  | |  | |  | |  | |  | |  | |  | |  | |  | |  | |  | |  | |  | |  | |  | |  | |  |  |
| 81 | Adopt BP | NI | Cattle | Control prog | V | Johne's disease | “On farm risk assessment” delivered by a vet in partnership with the farmer, seeking the proactive behaviour of the farmer and communication with the vet and supervision. | AH provider |  | |  | |  | |  | |  | |  | |  | |  | |  | |  | |  | |  | |  | |  | |  | |  | |  | |  | |  | |  | |  |  |
| 82 | Adopt BP | DK | General | Biosecurity | V | Biosecurity | Provision of information and training in meetings of different groups. | AH provider |  | |  | |  | |  | |  | |  | |  | |  | |  | |  | |  | |  | |  | |  | |  | |  | |  | |  | |  | |  | |  |  |
| 83 | Adopt BP | SE | Cattle | Control prog | V;C | BVD | "**Green list**" of BVD-free farms available to livestock traders. Easier to confirm status of herds prior to selling/buying, and to plan movements also used to see who not free - i.e. not present in the list. | Industry |  | |  | |  | |  | |  | |  | |  | |  | |  | |  | |  | |  | |  | |  | |  | |  | |  | |  | |  | |  | |  |  |
| 84 | Adopt BP | DK | Cattle | Biosecurity | V | Biosecurity | Nudging farms. Designed on the basis of nudging, using mainly **stimulus/priming strategies**: paths, colours, signs that lead to conduct an appropriate behaviour. | AH provider |  | |  | |  | |  | |  | |  | |  | |  | |  | |  | |  | |  | |  | |  | |  | |  | |  | |  | |  | |  | |  |  |
| 85 | Adopt BP | DK | Cattle | Control prog | V | Salmonella dublin | Herd categorisation determining trade and movements. | AH provider |  | |  | |  | |  | |  | |  | |  | |  | |  | |  | |  | |  | |  | |  | |  | |  | |  | |  | |  | |  | |  |  |
| 86 | Adopt BP | DK | Cattle | Control prog | V | Johne's disease | Herd categorisation determining trade and movements. | AH provider |  | |  | |  | |  | |  | |  | |  | |  | |  | |  | |  | |  | |  | |  | |  | |  | |  | |  | |  | |  | |  |  |
| 87 | Adopt BP | NI | Cattle | Control prog | V | Johne's disease | Herd categorisation determining trade and movements. | AH provider |  | |  | |  | |  | |  | |  | |  | |  | |  | |  | |  | |  | |  | |  | |  | |  | |  | |  | |  | |  | |  |  |
| 88 | Adopt BP | NO | Aquaculture | Control prog | C | Salmon lice | **Restriction:** if more 10 weeks higher levels of sea lice, **allowance (production) reduces** to half the next cycle and cannot be increased in other sites of the same producer. | Authorities |  | |  | |  | |  | |  | |  | |  | |  | |  | |  | |  | |  | |  | |  | |  | |  | |  | |  | |  | |  | |  |  |
| 89 | Adopt BP | DK | General | Biosecurity | V | Biosecurity | **Transport standard**: Cleaning + Disinfection of trucks. Use of washing facilities when returning from trade operations abroad. If an outbreak occurs in a farm due truck coming from an infected country, farmer covers it. | AH provider |  | |  | |  | |  | |  | |  | |  | |  | |  | |  | |  | |  | |  | |  | |  | |  | |  | |  | |  | |  | |  |  |
| 90 | Adopt BP | NL | Cattle | Biosecurity | V | unspecified | **Quality system**. dairy producers lose **points** if they acquire cattle from far sources. | Industry |  | |  | |  | |  | |  | |  | |  | |  | |  | |  | |  | |  | |  | |  | |  | |  | |  | |  | |  | |  | |  |  |
| 91 | Adopt BP | NL | Cattle | Control prog | V | Johne's disease | Milk not collected if seropositive animals are not culled. **Milk Quality Assurance**: Status A negative herds, B positive herds that have culled positive animals - C not culled, milk is not collected. | Industry |  | |  | |  | |  | |  | |  | |  | |  | |  | |  | |  | |  | |  | |  | |  | |  | |  | |  | |  | |  | |  |  |
| 92 | Enrol; Engage | SE | Cattle | Control prog | V | BVD | Industry requirements - participation in the BVD programme part of their quality programme. Only producers in the programme were allowed to deliver milk/animals to slaughter. Late stages. | Industry |  | |  | |  | |  | |  | |  | |  | |  | |  | |  | |  | |  | |  | |  | |  | |  | |  | |  | |  | |  | |  |  |
| 93 | Enrol; Engage | NL | Cattle | Control prog | C | leptospirosis | Milk is not collected if not part of the programme. | Industry |  | |  | |  | |  | |  | |  | |  | |  | |  | |  | |  | |  | |  | |  | |  | |  | |  | |  | |  | |  | |  |  |
| 94 | Engage | DK | General | AH management | V | General AH | Health Advisory Contract with the herd veterinarian= herd health programme - binding contract . | AH provider |  | |  | |  | |  | |  | |  | |  | |  | |  | |  | |  | |  | |  | |  | |  | |  | |  | |  | |  | |  | |  |  |
| 95 | Comply | CH | Cattle | Control prog | V | BVD | Linking BVD to the milk Quality assurance. | Industry |  | |  | |  | |  | |  | |  | |  | |  | |  | |  | |  | |  | |  | |  | |  | |  | |  | |  | |  | |  | |  |  |
| 96 | Adopt BP | SE | Cattle | Biosecurity | V | General AH | Quality requirement by the industry re import. | Industry |  | |  | |  | |  | |  | |  | |  | |  | |  | |  | |  | |  | |  | |  | |  | |  | |  | |  | |  | |  | |  |  |
| 97 | Adopt BP | SE | Cattle | Biosecurity | V | General AH | Quality requirement by the industry. | AH provider |  | |  | |  | |  | |  | |  | |  | |  | |  | |  | |  | |  | |  | |  | |  | |  | |  | |  | |  | |  | |  |  |
| 98 | Adopt BP | NO | Pigs | Biosecurity | V | General AH | Requirements by the industry – import. | Industry |  | |  | |  | |  | |  | |  | |  | |  | |  | |  | |  | |  | |  | |  | |  | |  | |  | |  | |  | |  | |  |  |
| 99 | Comply | NO | Pigs | Control prog | C | M hyopneumoniae | Close market access during eradication phase. | Industry |  | |  | |  | |  | |  | |  | |  | |  | |  | |  | |  | |  | |  | |  | |  | |  | |  | |  | |  | |  | |  |  |
|  |  | | | | | | | | | | | | | | | | | | | | | | | | | | | | | | | | | | | | | | | | | | | | | | | | | |
| In “**activity”**: control prog= control programme; in “**phase”**: V=voluntary phase, C= compulsory phase; in “**implementer”**: AH provider= Animal Health provider; *in* **“intervention ladder”**: 1= provision of information, 2=enable choice, 3= guide choice through a change in default policy, 4= Guide choice through the use of incentives, 5= Guide choice through the use of disincentives; in “**EAST”**: E= easy, S= social, A= attractive, T: timely, in “**MINDSPACE**”: M= messenger, I= Incentive, N= norms, D=default, S= salience, P= Priming, A=affect, C=commitment; E=ego. | | | | | | | | | | | | | | | | | | | | | | | | | | | | | | | | | | | | | | | | | | | | | | | | | | |
