# Supplementary material for: Nudging in Animal Disease Control and Surveillance: A Qualitative Approach to Identify Strategies Used to Improve Compliance With Animal Health Policies
Source: Front Vet Sci. 2020 Aug 5;7:383. doi: 10.3389/fvets.2020.00383 (PMC7419428; doi:10.3389/fvets.2020.00383)
Supplement: Supplementary file 2 [file Table_2.DOCX]

**Supplementary File 2**. Characteristics of strategies excluded from the Nuffield ladder, aiming to make the system more efficient.

| **Desired behaviour** | **Country** | **Species** | **Activity** | **Phase** | **Disease/ pathogen** | **Description** | **Implementer** | **E** | **A** | **S** | **T** | **M** | **I** | **N** | **D** | **S** | **P** | **A** | **C** | **E** |
| --- | --- | --- | --- | --- | --- | --- | --- | --- | --- | --- | --- | --- | --- | --- | --- | --- | --- | --- | --- | --- |
| Accept surveillance | SE | Cattle | Control prog | C | BVD | Sampling was shifted from the farm to slaughterhouse to reduce resistance to testing, as cumbersome for beef farmers expensive. Reduced costs. | Industry |  |  |  |  |  |  |  |  |  |  |  |  |  |
| Accept surveillance | SE | Cattle | Control prog | C | BVD | Rolling subscriptions for bulk milk testing to have the BVD status of dairy herds regularly updated, requirement for selling animals as BVD-free. | Industry |  |  |  |  |  |  |  |  |  |  |  |  |  |
| Accept surveillance | IE | Cattle | Control prog | V | BVD | Tissue tag testing. Convenience and cut down the costs. - existence of arrangement with the labs that streamline the payment process and discounts. | Industry |  |  |  |  |  |  |  |  |  |  |  |  |  |
| Accept surveillance | NO | Cattle | Control prog | C | Johne's disease | Sampling exclusion of producers sampled the previous year ( or year before) from the total to be sampled, for one or two years. ( JD is notifiable) | Authorities |  |  |  |  |  |  |  |  |  |  |  |  |  |
| Accept surveillance | NO | Pigs | Control prog | C | General | After eradication make disease notifiable so that industry do not have to bear the responsibility after having funded programmes for years. | Authorities |  |  |  |  |  |  |  |  |  |  |  |  |  |
| Enrol; Engage | DK | Cattle | Control prog | V | Johne's disease | Funding to achieve better DX tests - intended strategy - to achieve better Dx test | AH provider |  |  |  |  |  |  |  |  |  |  |  |  |  |
| Enrol; Engage | DK | Cattle | Control prog | V | Johne's disease | Investigation of different transmission routes to be addressed by specific and effective measures - intended strategy - to achieve better control strategy | AH provider |  |  |  |  |  |  |  |  |  |  |  |  |  |
| Enrol; Engage | NI | General | Control prog | V | BVD | Regular meetings of authorities, promote good communication between local authorities and slaughterhouses, others. “all island technical group” | AH provider |  |  |  |  |  |  |  |  |  |  |  |  |  |
| Enrol; Engage | NI | Cattle | AH management | V | Johne's disease | “Repackaging” of the RA - Change the on farm risk assessment- to be part of a biosecurity cow-health package that includes key elements of on farm RA. | AH provider |  |  |  |  |  |  |  |  |  |  |  |  |  |
| Engage | NI | General | AH management | V | General AH | Meetings between AHNI and authorities every 2-3 weeks, promote good communication between local authorities and slaughterhouses, others Existence of an “all island technical group” | AH provider |  |  |  |  |  |  |  |  |  |  |  |  |  |
| Engage | NE | Pigs | control prog | C | Salmonella spp. | If Salmonella positive for long time, need to get in touch with a person to work towards to seronegative status | Industry |  |  |  |  |  |  |  |  |  |  |  |  |  |
| Engage | NO | Aquaculture | AH management | V | General AH | Creation of Economic Funds to cover some diseases (e.g. Pancreatic disease) | *Authorities; Industry* |  |  |  |  |  |  |  |  |  |  |  |  |  |
| Comply | DK | General | AH management | V | General AH | Meetings to provide information and enhance relationships and trust. | Authorities |  |  |  |  |  |  |  |  |  |  |  |  |  |
| Comply | CH | Cattle | Control prog | C | BVD | Sampling transferred to slaughterhouses | Authorities |  |  |  |  |  |  |  |  |  |  |  |  |  |
| Comply | NO | Cattle | Control prog | C | Salmonella spp. | Convenient and flexible sampling system instead of strict random. Aiming to involve the samplers in the system actively and making them understand the objectives and purpose of the surveillance strategy. | Authorities |  |  |  |  |  |  |  |  |  |  |  |  |  |
| Comply | NO | Cattle | Surveillance | C | Scrapie |  | Authorities |  |  |  |  |  |  |  |  |  |  |  |  |  |
| Comply | NO | Cattle | Surveillance | C | BVD; IBR; EBL | To proof freedom of these diseases - lists were provided to the authorities (NFSA) to facilitate the task | Authorities |  |  |  |  |  |  |  |  |  |  |  |  |  |
| Comply | NO | Pigs | Control prog | C | MRSA | Eradication. Reports on the disease occurrence (suspicion of disease, etc.) are sent to authorities 🡪 industry stakeholders | Authorities |  |  |  |  |  |  |  |  |  |  |  |  |  |
| Comply | NO | Pigs | Control prog | C | MRSA | Eradication - Compensation for the depopulation - national funds at county level: some direct costs and losses. | Authorities |  |  |  |  |  |  |  |  |  |  |  |  |  |
| Comply | NO | Aquaculture | Surveillance | C | ISA;Notifiable | Promote responsible fish health services that trust in authorities and their decisions, to promote population health. | AH provider |  |  |  |  |  |  |  |  |  |  |  |  |  |
| Adopt BP | NO | Aquaculture | AH management | V | General AH | Producers' and industry demands generate bigger pressure than authorities. | Industry |  |  |  |  |  |  |  |  |  |  |  |  |  |
| In “**activity”**: control prog= control programme; in “**phase”**: V=voluntary phase, C= compulsory phase; in “**implementer”**: AH provider= Animal Health provider; *in* **“intervention ladder”**: 1= provision of information, 2=enable choice, 3= guide choice through a change in default policy, 4= Guide choice through the use of incentives, 5= Guide choice through the use of disincentives; in “**EAST”**: E= easy, S= social, A= attractive, T: timely, in “**MINDSPACE**”: M= messenger, I= Incentive, N= norms, D=default, S= salience, P= Priming, A=affect, C=commitment; E=ego. | | | | | | | | | | | | | | | | | | | | |
