# Supplementary material for: Nudging in Animal Disease Control and Surveillance: A Qualitative Approach to Identify Strategies Used to Improve Compliance With Animal Health Policies
Source: Front Vet Sci. 2020 Aug 5;7:383. doi: 10.3389/fvets.2020.00383 (PMC7419428; doi:10.3389/fvets.2020.00383)
Supplement: Supplementary file 3 [file Data_Sheet_1.docx]

**Supplementary file 3. Overview of strategies in Supplementary File 1 and 2.**

### Strategies that presented less than three EAST attributes

Among the 65 strategies that had two attributes or less from the EAST framework, 24 were classified as guiding choice through incentives, and 8 were guiding choice through disincentives, based on the Nuffield ladder. The use of incentives, as psychological mechanism defined in MINDSPACE, was identified in 42 strategies (Supplementary file 1). These strategies were very diverse, both in relation to the behaviour to influence as well as the type of activity, or stage of a programme they were applied in. Monetary incentives (payments, rewards) are common. However, we found that a number of the strategies in this study had indeed non-monetary incentives, such as free access to attractive information or services (strategies no.56-62 in Supplementary file 1). Authorities in DK have designed systems to incentivise producers to submit samples in exchange for valuable information, such as results of other diseases of interest (like Trichinella) or more in-depth molecular analysis on mastitis, or choice of vaccines for swine influenza infections. Similarly, in NL, reports and feedback are provided in aquaculture. In NO, surveillance of diseases absent in the country, such as *B. abortus,* is combined with analyses for other diseases of interest to producers, such as Toxoplasma, to incentivise the submission of samples. These strategies make the submission of samples more *Attractive* (strategies no.60,61, Supplementary file 1).

Financial incentives, often in combination with other strategies, were used when aiming for enrolment and compliance with a control or biosecurity programme. In two examples (strategies no. 37,38 Supplementary 1), affiliated herds would receive a higher economic compensation for costs caused by the restrictions and requirements applied within the Swedish *Salmonella* control programme, if *Salmonella* infection is detected in the herd. There were also other strategies described to enhance the engagement in the program, such as the use of an interactive IT system to engage farmers. In the first level (of three) of the programme the aim is to increase engagement using *Attractive* and *Social* design features. A main psychological mechanism in this respect is *Commitment* since, although voluntary, the programme involves a formal subscription and payment of a fee. Farmers then assess their own routines and practices using a questionnaire, the farmer is then provided with the results by means of a traffic light system. In combination with this questionnaire, an online information platform is available, with videos of other farmers explaining their experiences and views, animations on salient biosecurity risks, and other material to attract the farmer’s interest in programme progression. The first stage has a credible *Messenger*, informs about what others do (*Norms*), acts on *Salient* beliefs and generates (possibly) *Affect*, involves *Commitment* and boosts farmer’s *Ego*. In this way, programme progression becomes attractive for farmers, even when requiring more engagement, costs and also involvement of a veterinarian.

### Strategies outside of the Nuffield ladder

Information was also collected on 21 strategies that were excluded from classification by the Nuffield ladder. The common feature was that they acted at a systems level with the aim to make it more efficient (Supplementary file 2). Despite not being intended to influence a certain behaviour in an individual, potential indirect effects were identified, and in some of them, features were compatible with attributes and mechanisms of EAST and MINDSPACE. These strategies mostly addressed acceptance of surveillance once an infection had been controlled or eradicated by using *Default* designs. One example of this was shifting sampling from farms to abattoirs once BVD was controlled in SE, and thereby reducing the potential resistance for testing of beef cattle that would otherwise have to be tested on farm. Another example was a strategy in NO concerning surveillance for proving freedom from disease, where herds testing negative are excluded from sampling in the next two years, thus reducing the farmer’s effort. A third example is from NI in the early phase of a control programme for paratuberculosis. Instead of aiming for the implementation of a full program, certain key elements such as calf management, were included in a biosecurity cow-health package, believed to “attract the interest of producers and avoiding burdening them with another programme”. In other words, these strategies did not aim at influencing individual decisions to enrol, engage or conform; rather they were overarching strategies aimed at reducing the surveillance and control burden for all farmers covered by these activities.
